# Natural Selection of *ATP2B1* Underlies Susceptibility to Essential Hypertension

**DOI:** 10.3389/fgene.2021.628516

**Published:** 2021-03-11

**Authors:** Lin-dan Ji, Zhi-feng Xu, Nelson L. S. Tang, Jin Xu

**Affiliations:** ^1^Department of Biochemistry, School of Medicine, Ningbo University, Ningbo, China; ^2^Zhejiang Key Laboratory of Pathophysiology, School of Medicine, Ningbo University, Ningbo, China; ^3^Department of Cardiology, Ningbo No. 7 Hospital, Ningbo, China; ^4^Department of Chemical Pathology, Faculty of Medicine, Li Ka Shing Institute of Health Sciences, The Chinese University of Hong Kong, Hong Kong, China; ^5^KIZ-CUHK Joint Laboratory of Bio-resources and Molecular Research of Common Diseases, The Chinese University of Hong Kong, Hong Kong, China; ^6^Department of Preventive Medicine, School of Medicine, Ningbo University, Ningbo, China

**Keywords:** essential hypertension, ATP2B1, single nucleotide polymorphism, natural selection, genome-wide association study

## Introduction

The high prevalence of essential hypertension and its uneven distribution across different populations is both a major public health concern and a puzzle in evolutionary biology (Rossier et al., 2017). Why is this deleterious disease so common when the causal variants are expected to be selected against by natural selection? Several hypotheses have been put forth to explain this paradox. Most of them hypothesize that in the past, these variants conferred an evolutionary advantage to our ancestors. The “thrifty genotype” hypothesis (Neel, 1962) and the “sodium and water retention” hypothesis (Young et al., 2005) propose that the risk factors for hypertension, such as enhanced salt and water avidity and vascular reactivity, are adaptive traits associated with salt scarcity and the hot and humid climate of the ancestral African environment (Gleibermann, 1973; Young et al., 2005). As humans migrated out of Africa to cooler climates, the genes originally selecting for hot and humid climates and sodium conservation became maladaptive for today's environment, and individuals with these ancient genotypes could have an increased risk for developing hypertension (Weder, 2007). Therefore, it has been suggested that variability in hypertension susceptibility is due to selection in response to the various climates encountered during the out-of-Africa expansion (Young et al., 2005).

Three hypertension susceptibility genes—*AGT, CYP3A5* and *GNB3*—have been suggested to have undergone natural selection (Nakajima et al., 2004; Thompson et al., 2004; Young et al., 2005), providing a new way to study genetic susceptibility to hypertension. Recently, we carried out a systematic evolutionary analysis on the six human renin-angiotensin-aldosterone system (RAAS) genes—*ACE, AGT, AGTR1, AGTR2, CYP11B2*, and *REN*—and found that five single nucleotide polymorphisms (SNPs) within *AGTR1* underwent natural selection in Euro-Asian populations in relation to ambient temperature. Among these SNPs, only rs1873902 differed significantly between hypertensive patients and normotensive controls after Bonferroni correction. The risk allele was shown to be the ancestral African dominant allele (Ji et al., 2016). These studies strongly support the “thrifty genotype” hypothesis and the “sodium and water retention” hypothesis, which state that the genes originally selected for effective heat dissipation and sodium conservation are maladaptive in the present environment and could increase susceptibility to hypertension.

## Genome-Wide Association Studies Identified *ATP2B1* is a Susceptibility Gene for Essential Hypertension

Considering the relatively small sample sizes, biased study designs, and low genetic power, the results from candidate gene approach studies are usually unreliable. After dozens of GWASs on hypertension, *ATP2B1* is the first gene to have been cross-validated in different GWASs. In 2009, a GWAS conducted by the Cohorts for Heart and Aging Research in Genome Epidemiology (CHARGE) consortium found that *ATP2B1* genetic polymorphisms were significantly related to systolic blood pressure (SBP), diastolic blood pressure (DBP), and hypertension (Levy et al., 2009). These SNPs were replicated in European populations by the Global Blood Pressure Genetics (Global BPgen) Consortium (Newton-Cheh et al., 2009), and also in Asian populations by the Korean Association Resource (KARE) (Cho et al., 2009), the Japanese Millennium Genome Project (Tabara et al., 2010), the Genetic Epidemiology Network of Cardiovascular Disease in China (GENECDC) (Lu et al., 2015), and other Asian cohorts(Takeuchi et al., 2018). Moreover, combined analysis of the two largest cohorts, CHARGE and Global BPgen, further confirmed that only *ATP2B1* variants were able to reach genome-wide significance (*P* < 5 × 10^−8^) with SBP (rs2681492), DBP (rs2681472) and hypertension (rs2681472) (Hirawa et al., 2013).

## Evolutionary Analysis on the *ATP2B1* Gene

The aim of this study was to gain further insights into the relationship between climate adaptation and hypertension and to determine why, from an evolutionary point of view, *ATP2B1* became a susceptibility gene for hypertension. To answer these questions, we carried out an evolutionary analysis on the *ATP2B1* gene. The SNPs showing natural selection signals were further analyzed to determine the potential driving force for selection. Finally, the SNPs were examined in a sample Chinese population with essential hypertension. The methods have been described previously (Ji et al., 2016, in press).

**Figure 1:**
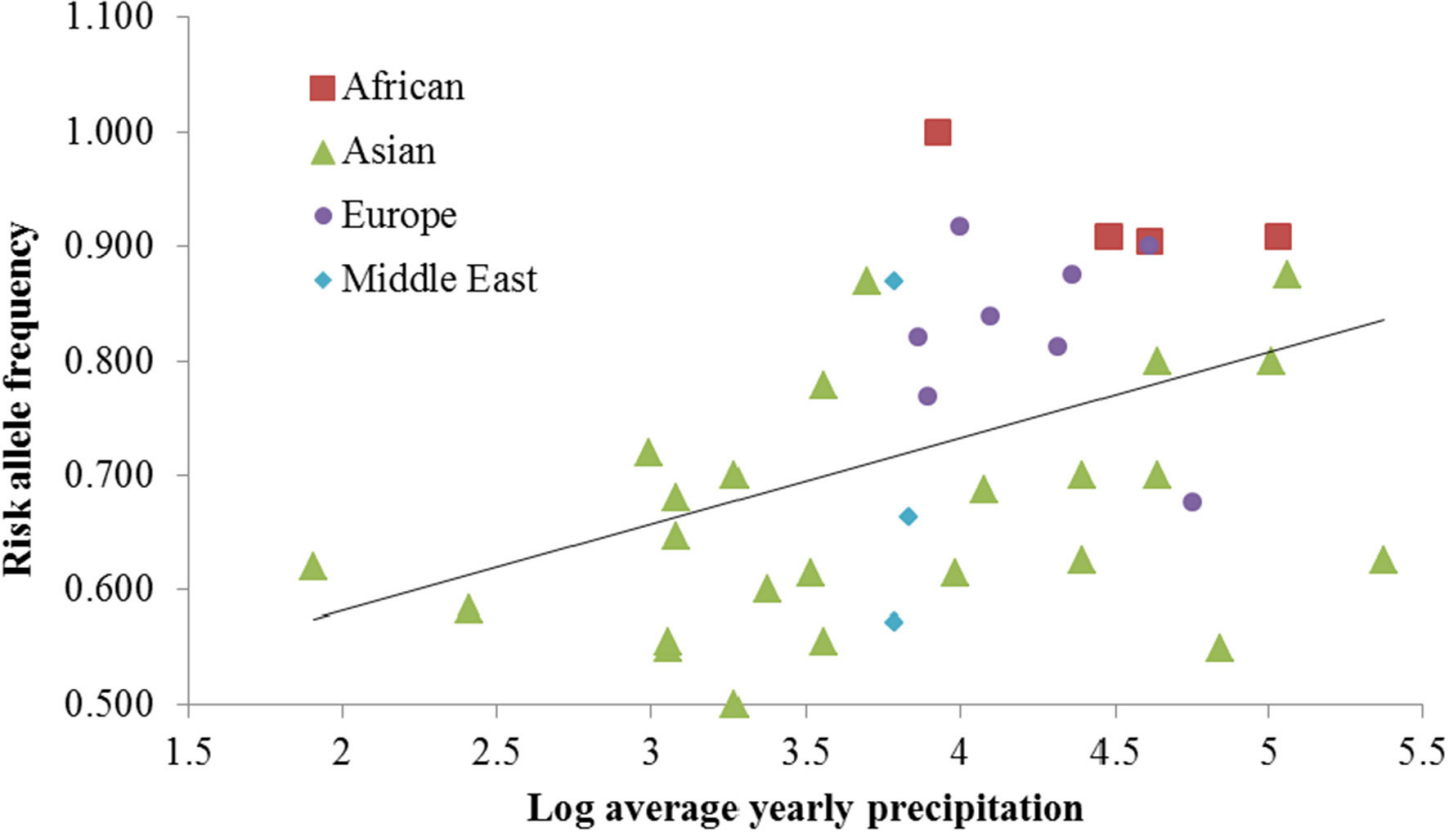
The risk T allele of rs2681472 is strongly associated with precipitation among CEPH-HGDP populations.

All 87 SNPs in the *ATP2B1* gene were retrieved from the CEPH-HGDP dataset (Li et al., 2008). Two selective parameters—integrated haplotype score (iHS) and Wright's fixation index (*F*_ST_)—were used to screen candidate SNPs that had undergone natural selection. After iHS analysis, three SNPs (rs10745527, rs11105550, and rs1438993) showed positive selection signals in the ASN population, two SNPs (rs11105404 and rs2681472) in the YRI population, and none in the CEU population (|iHS|>2). Further *F*_ST_ analysis of the CEPH-HGDP data indicated that only rs2681472 was shown to be significantly different between ASN and YRI populations (*F*_ST_=0.20). The other four SNPs did not differ between any two populations. To determine the potential driving force for selection, we subsequently performed a correlation analysis between the derived allele frequency and daily sunshine duration, temperature, ultraviolet radiation and precipitation using correlation analysis. The effects of these environmental factors, along with the geographic parameters, such as longitude, latitude and altitude, were evaluated using multiple linear regression analysis. Both the bivariate correlation analysis and the multiple linear regression analysis revealed that rs2681472 was significantly associated with precipitation (*R* = 0.431, *P* = 0.006, Figure 1). We also searched the dbCLINE (Hancock et al., 2011), a software of genome-wide scan for evidence of positive selection in response to climatic variation, and it also indicated that rs2681472 is associated with summer precipitation rate. Because only rs2681472 exhibited a positive selection signal, it was further genotyped by Tm-shift method (Yuan et al., 2012) in 2032 individuals from a sample Chinese population. The 1,016 essential hypertension patients and 1,016 healthy participants were recruited in Ningbo, a city in east China, and the case and control were well-matched for age and sex. The result revealed significant differences between the case and control groups (*P* = 0.002, OR=1.23, 95% CI=1.08–1.39). The risk allele T was shown to be the ancestral African dominant allele.

## Discussion

Based on the GWASs and the following replication studies, the SNP rs2681472 of the *ATP2B1* gene was confirmed to be associated with blood pressure or hypertension in various populations (Levy et al., 2009; Newton-Cheh et al., 2009; Hirawa et al., 2013; Kato et al., 2015; Nikpay et al., 2015; Liu et al., 2016; Nelson et al., 2017; Takeuchi et al., 2018). The current study suggests that *ATP2B1* became a susceptibility gene for hypertension via natural selection of this SNP. Moreover, according to the Genotype-Tissue Expression (GTEx) project, although rs2681472 is an intron SNP, it is a typical expression quantitative trait loci (eQTL) in multi-tissues, including artery and aorta (Consortium, 2020). Therefore, rs2681472 is not only a tagSNP, but also a functional variant have undergone natural selection.

The *ATP2B1* gene encodes the plasma membrane calcium ATPase isoform 1, and results from *ATP2B1* knockout mouse studies suggest that *ATP2B1* may play an important role in the regulation of BP through alterations in calcium handling and vasoconstriction of vascular smooth muscle cells (Kobayashi et al., 2012). Because heat dissipation through sweating results in large volume losses, further enhancement of vasoconstriction was likely part of our adaptation to the hot and humid African environment. As our ancestors migrated out of Africa, the primary thermodynamic requirement shifted from heat dissipation to heat conservation. Selection for salt and water avidity and vasoconstriction lessened. This difference in volume avidity and vasoconstriction, an important physiologic source of hypertension susceptibility, may be a consequence of differential exposure to selection pressures since the out-of-Africa expansion.

## Author Contributions

L-dJ and JX conceived the opinion. L-dJ and Z-fX completed the evolutionary analysis and case-control replication study. L-dJ, NT, and JX wrote the manuscript. All authors contributed to the article and approved the submitted version.

## Conflict of Interest

The authors declare that the research was conducted in the absence of any commercial or financial relationships that could be construed as a potential conflict of interest.
